# Supplementary material for: The properties of human disease mutations at protein interfaces
Source: PLoS Comput Biol. 2022 Feb 4;18(2):e1009858. doi: 10.1371/journal.pcbi.1009858 (PMC8849535; doi:10.1371/journal.pcbi.1009858)
Supplement: S1 Text — Fig A in S1 Text: Enrichment of pathogenic variants at cognate and non-cognate ligand interfaces. Error bars represent 95% confidence intervals. Enrichment values shown are relative to the full dataset. Detailed relative enrichment data is available in S1 Table. Fig B in S1 Text: The impact of ‘benign’ dataset on the enrichment of pathogenic mutations. Relative enrichment of pathogenic mutations using common (≥1%) gnomAD variants, rare (<1%) gnomAD variants and ClinVar benign and likely benign variants in: A) different locations (surface, interior and interface regions); B) different interface regions (core, support and rim); and C) different protein interface types. All error bars represent 95% confidence intervals. Detailed relative enrichment data is available in S1 Table. Fig C in S1 Text: Enrichment of pathogenic variants at different protein locations and interface types when excluding predicted membrane proteins. A) Enrichment of pathogenic variants in surface, interior and interface locations relative to all data. B) Enrichment of pathogenic variants in different interface types relative to all data. Total numbers of pathogenic and putatively benign variants are shown below the plots. Error bars represent 95% confidence intervals. Detailed relative enrichment data is available in S1 Table. Fig D in S1 Text: The impact of protein biological function on the enrichment of pathogenic mutations. A) The enrichment of pathogenic mutations at different locations and interface types for proteins annotated as having/lacking catalytic activity in Uniprot. B) The enrichment of pathogenic mutations at different locations and interface types for proteins annotated as being/not being involved in transcriptional regulation in Uniprot. C) The enrichment of pathogenic mutations at different locations and interface types for proteins annotated as having/lacking transporter activity in Uniprot. All error bars represent 95% confidence intervals. Detailed relative enrichment data [file pcbi.1009858.s001.docx]

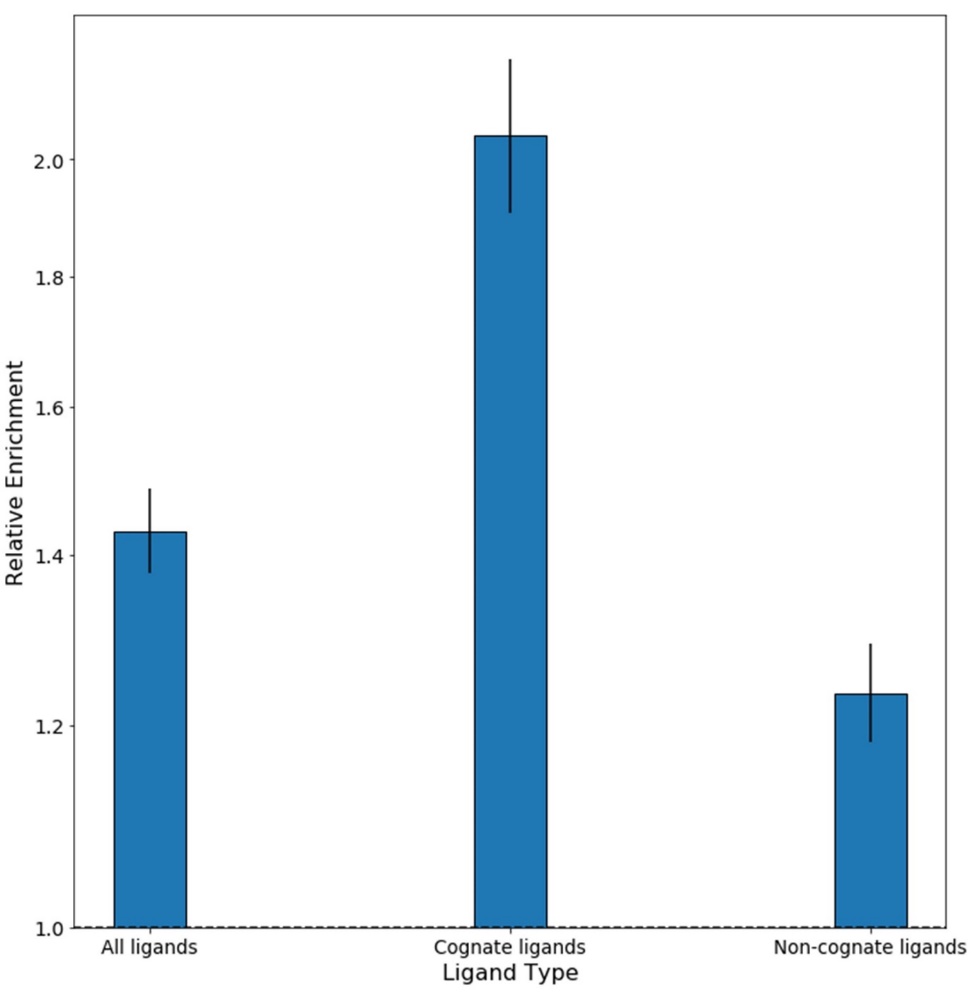


**Figure A: Enrichment of pathogenic variants at cognate and non-cognate ligand interfaces.** Error bars represent 95% confidence intervals. Enrichment values shown are relative to the full dataset. Detailed relative enrichment data is available in S1 Table.


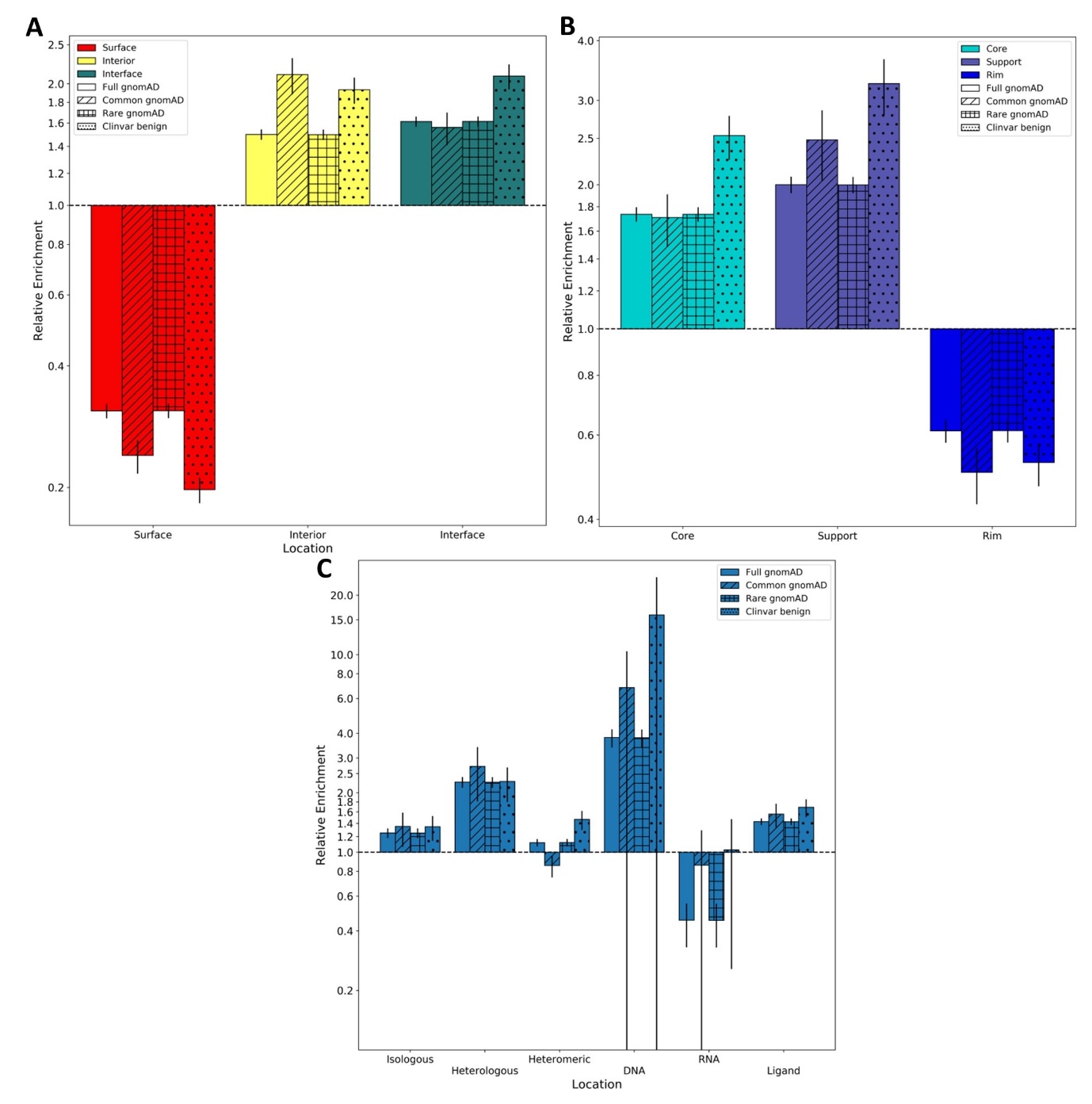


**Figure B: The impact of ‘benign’ dataset on the enrichment of pathogenic mutations.** Relative enrichment of pathogenic mutations using common (≥1%) gnomAD variants, rare (<1%) gnomAD variants and ClinVar benign and likely benign variants in: A) different locations (surface, interior and interface regions); B) different interface regions (core, support and rim); and C) different protein interface types. All error bars represent 95% confidence intervals. Detailed relative enrichment data is available in S1 Table.


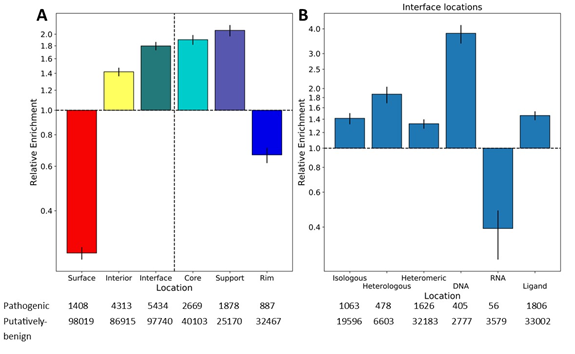


**Figure C: Enrichment of pathogenic variants at different protein locations and interface types when excluding predicted membrane proteins.** A) Enrichment of pathogenic variants in surface, interior and interface locations relative to all data. B) Enrichment of pathogenic variants in different interface types relative to all data. Total numbers of pathogenic and putatively benign variants are shown below the plots. Error bars represent 95% confidence intervals. Detailed relative enrichment data is available in S1 Table.


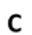

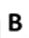

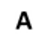

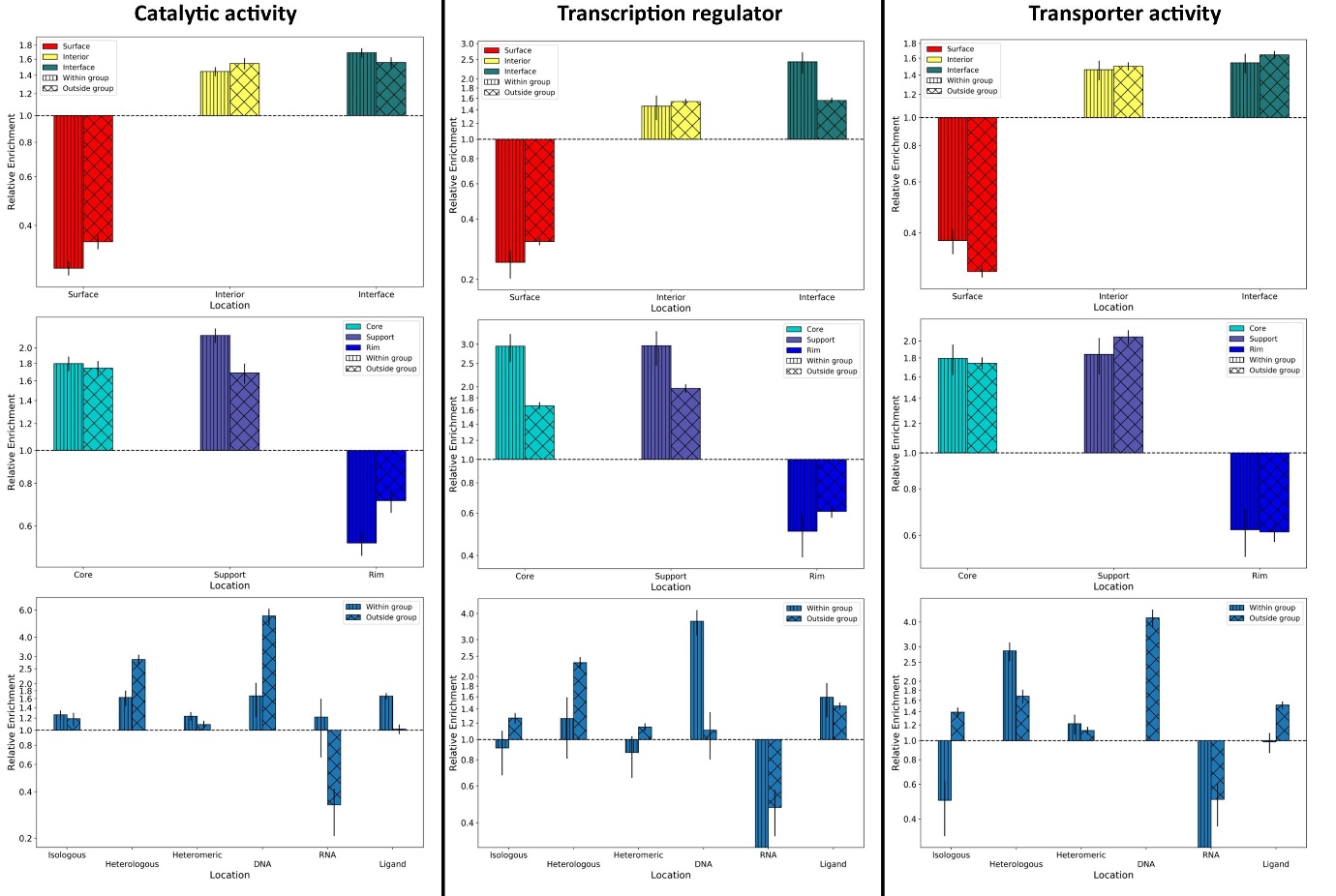


**Figure D: The impact of protein biological function on the enrichment of pathogenic mutations.** A) The enrichment of pathogenic mutations at different locations and interface types for proteins annotated as having/lacking catalytic activity in Uniprot. B) The enrichment of pathogenic mutations at different locations and interface types for proteins annotated as being/not being involved in transcriptional regulation in Uniprot. C) The enrichment of pathogenic mutations at different locations and interface types for proteins annotated as having/lacking transporter activity in Uniprot. All error bars represent 95% confidence intervals. Detailed relative enrichment data is available in S1 Table.

**
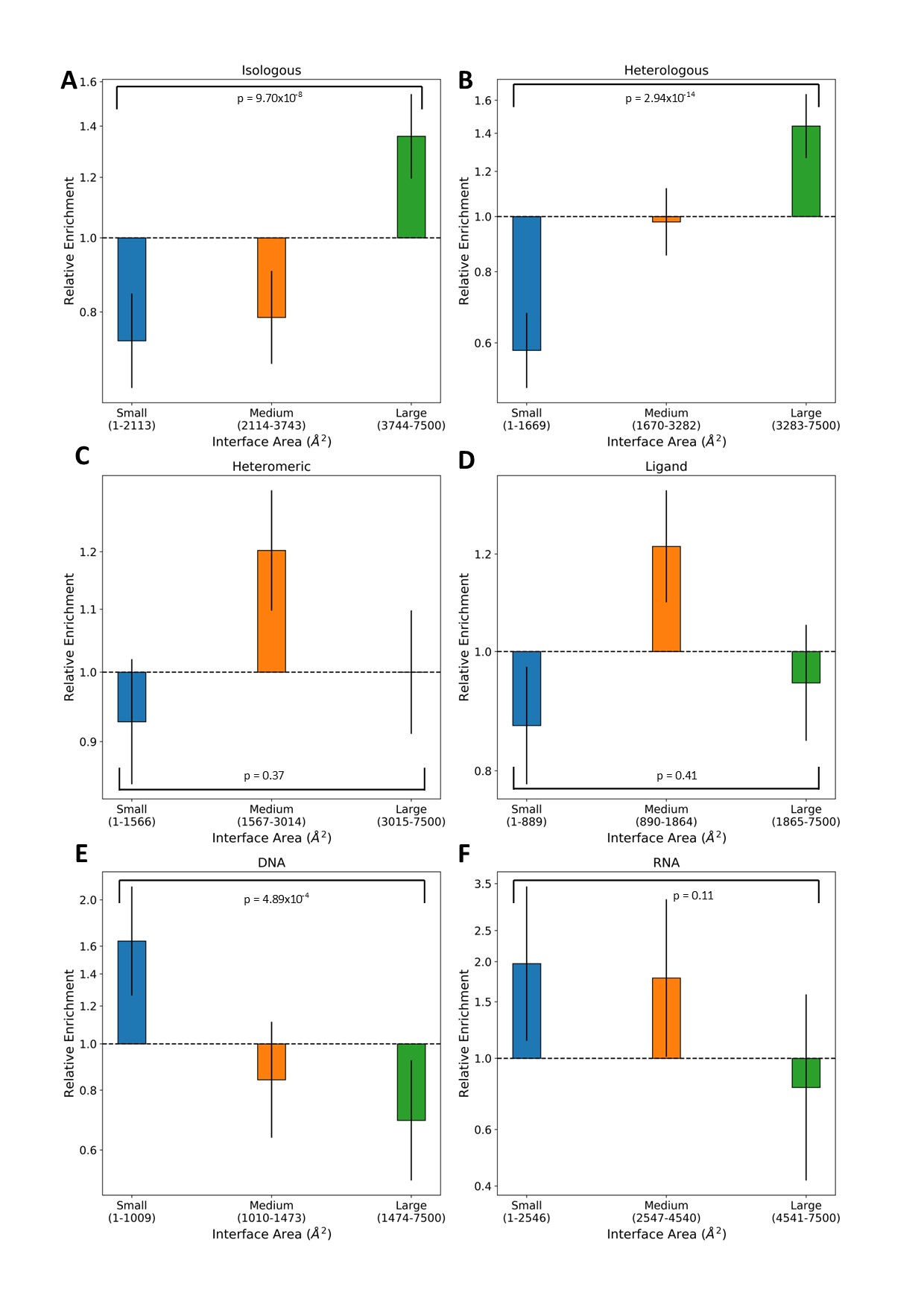
**

**Figure E: The impact of interface size on the enrichment of pathogenic variants.** Enrichment of pathogenic variants within interface residues from interfaces of different sizes. Each interface type was split into three groups of small, medium and large interfaces, containing equal numbers of variants. A) Homomeric isologous; B) homomeric heterologous; C) heteromeric; D) ligand; E) DNA; and F) RNA interface residues. Error bars represent 95% confidence intervals. Detailed relative enrichment data is available in S1 Table.


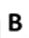

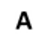

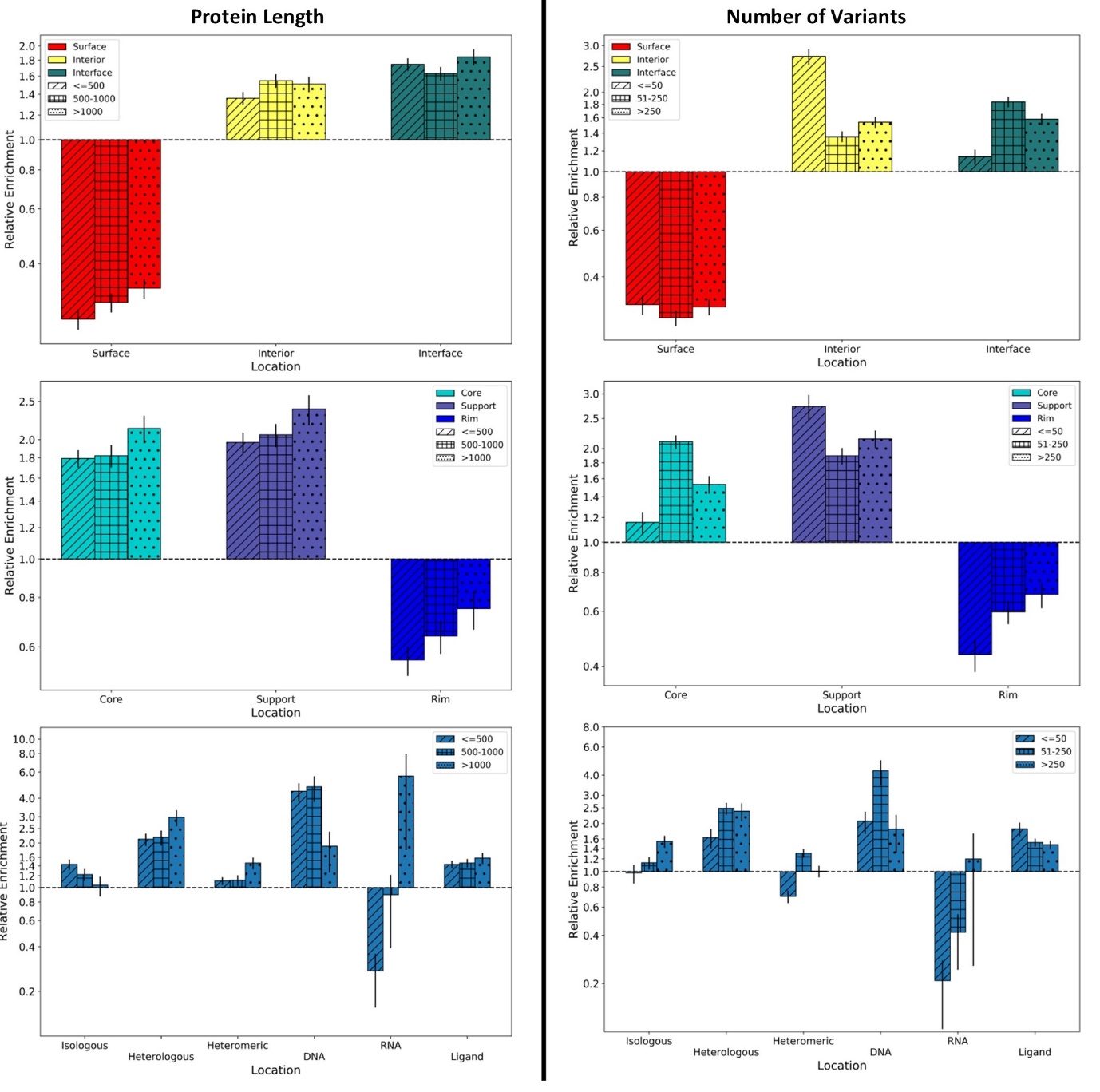


**Figure F: The impact of protein length and variant number on the enrichment of pathogenic mutations.** A) The enrichment of pathogenic mutations at different locations and interface types for proteins of different amino acid chain lengths (<=500 residues, 500-1000 residues, >1000 residues). B) The enrichment of pathogenic mutations at different locations and interface types for proteins with different numbers of variants in our datasets (combined ClinVar and gnomAD). All error bars represent 95% confidence intervals. Detailed relative enrichment data is available in S1 Table.


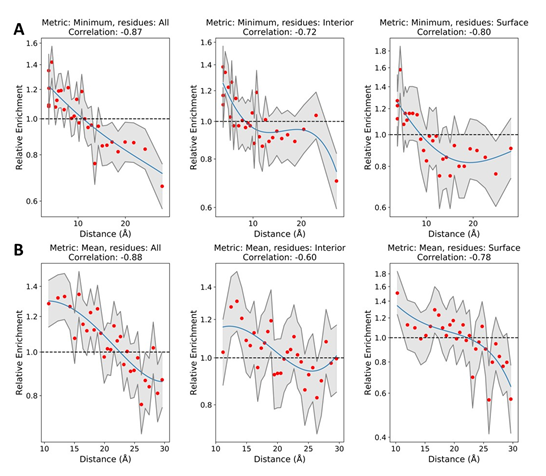


**Figure G: Odds ratio of disease mutations at increasing distance from the nearest interface.** A) Using the minimum distance to the nearest interface residue. B) Using the mean distance to all interface residues. 95% confidence intervals are shown in grey, a univariate spline (blue) has been fit to the data. Charts are shown for surface and interior residues together and individually.


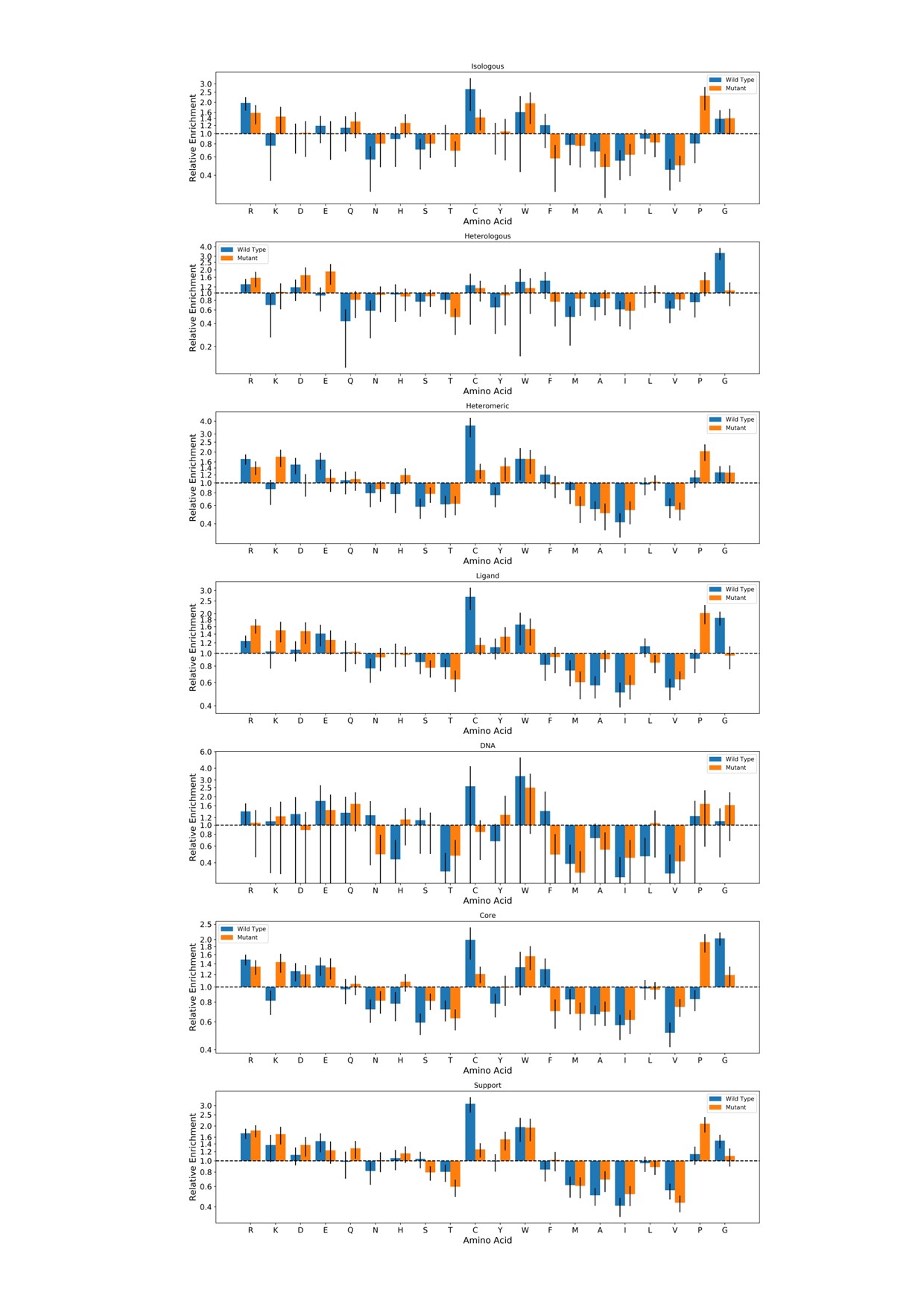


**Figure H: Enrichment of pathogenic variants involving different amino acid residues for different types of interfaces.** The odds ratio of pathogenic mutations associated with specific amino acid mutations at each interface type as well as core and support regions. Blue bars represent mutations *from* a specific amino acid, while orange bars represent mutations *to* a specific amino acid. Error bars represent 95% confidence intervals. Detailed relative enrichment data is available in S1 Table.


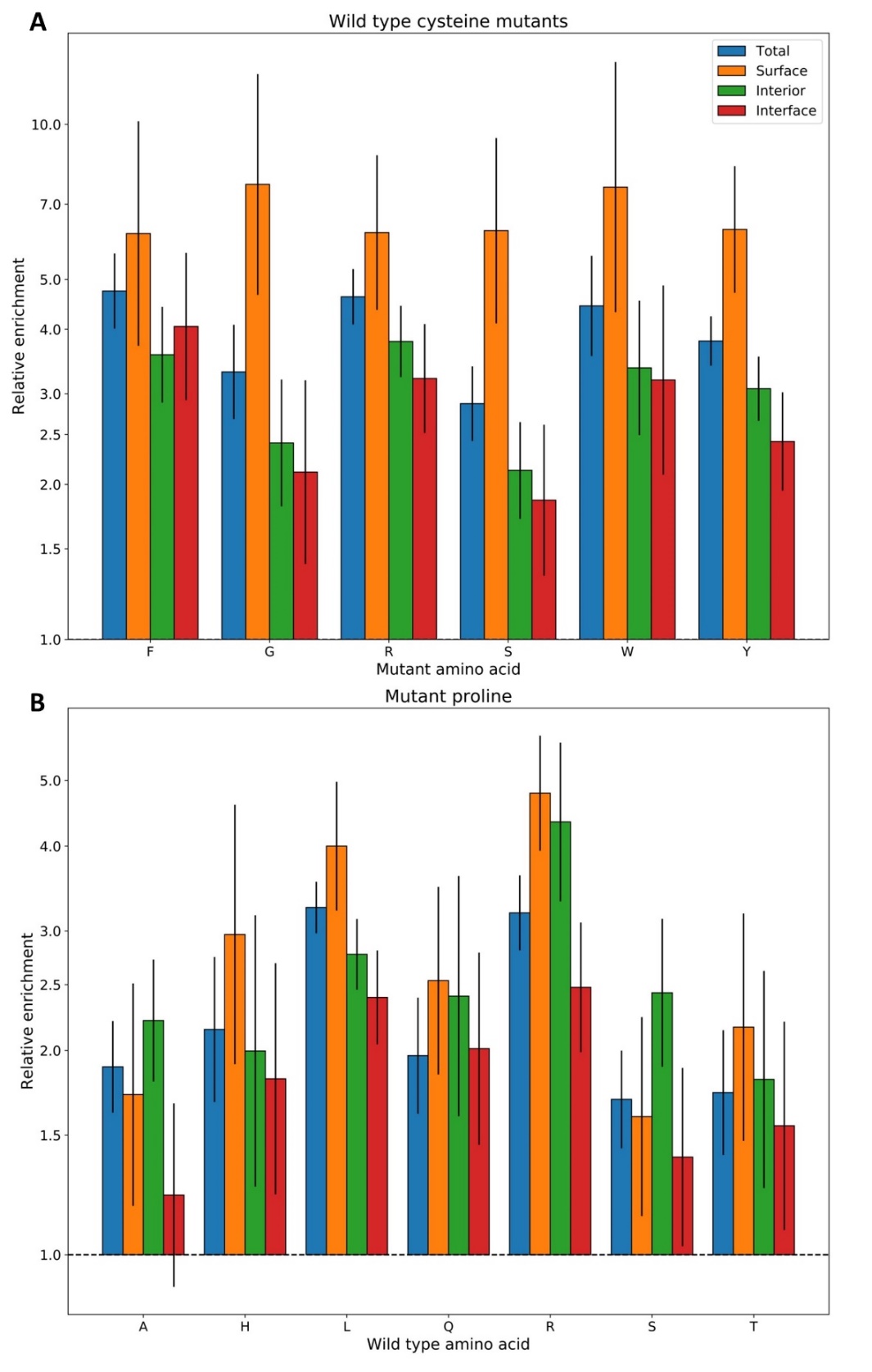


**Figure I: Relative enrichment of pathogenic variants for cysteine and proline mutants.** A) Enrichment of pathogenic variants for all mutations possible via a single nucleotide change from wild-type cysteine at different protein locations. B) Enrichment of pathogenic variants for all wild-type amino acids that could mutate to proline via a single nucleotide change. All error bars represent 95% confidence intervals. Detailed relative enrichment data is available in S1 Table.


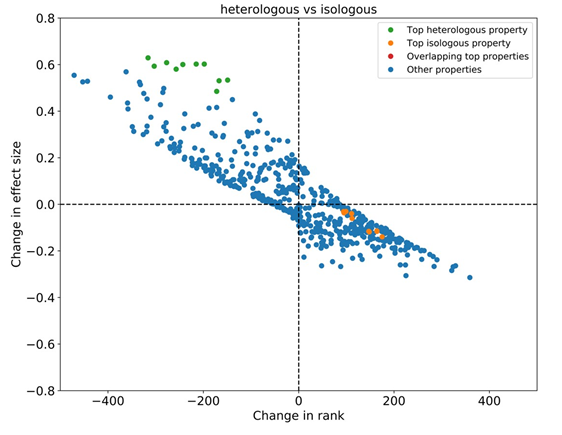


**Figure J: Difference in property changes between isologous and heterologous interfaces plotted against rank change of those properties with all predicted membrane proteins excluded.** Effect size difference in the delta properties of two locations is on the Y-axis, and rank change of the delta properties is on the X-axis. The top properties (by effect size) for each location are coloured green and orange.


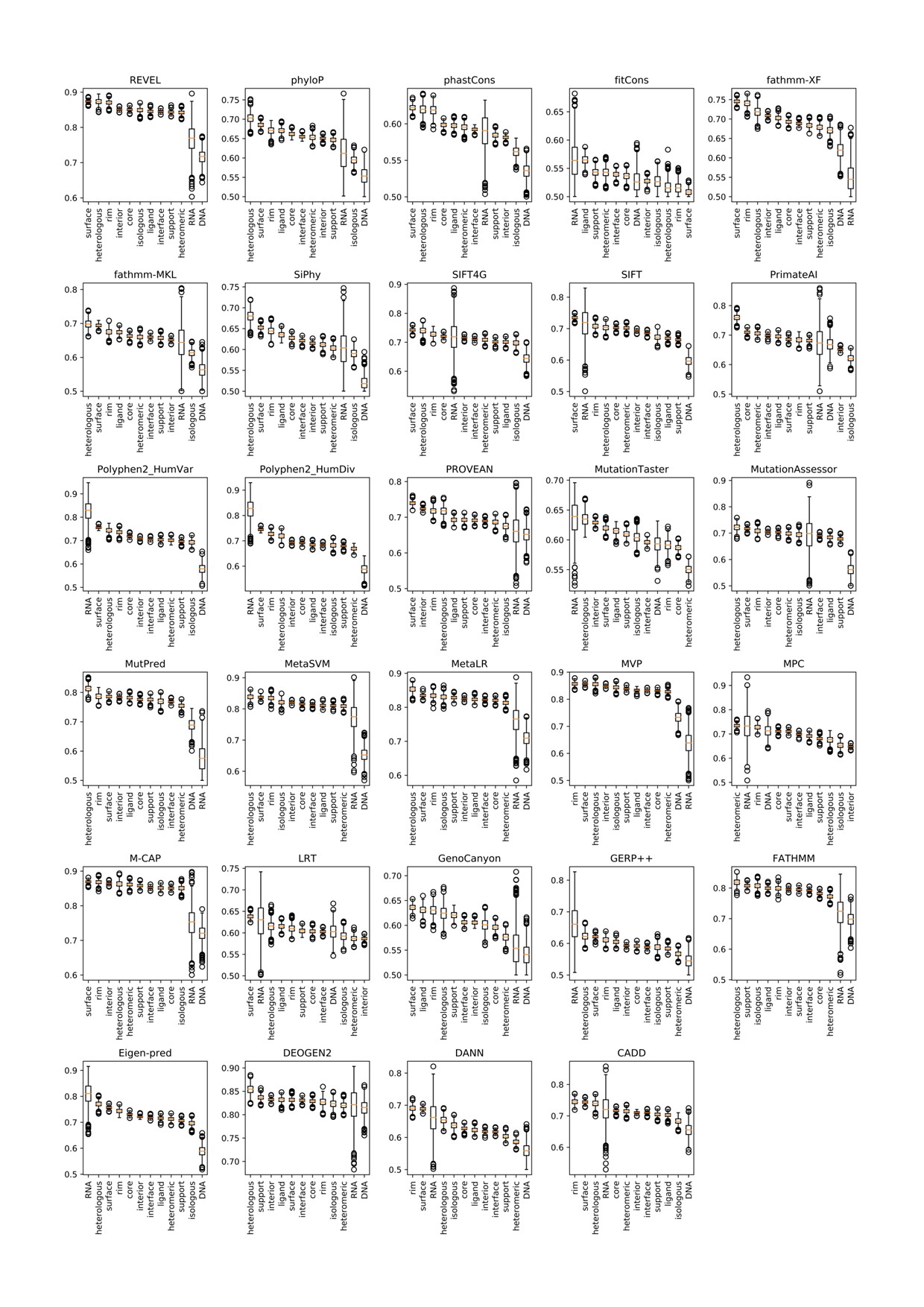


**Figure K: Distribution of bootstrapped ROC AUC for predictions made by different computational methods.** Pathogenic and putatively benign datasets were independently re-sampled 1000 times with replacement. Only mutations with all 29 predictions were included in this analysis.


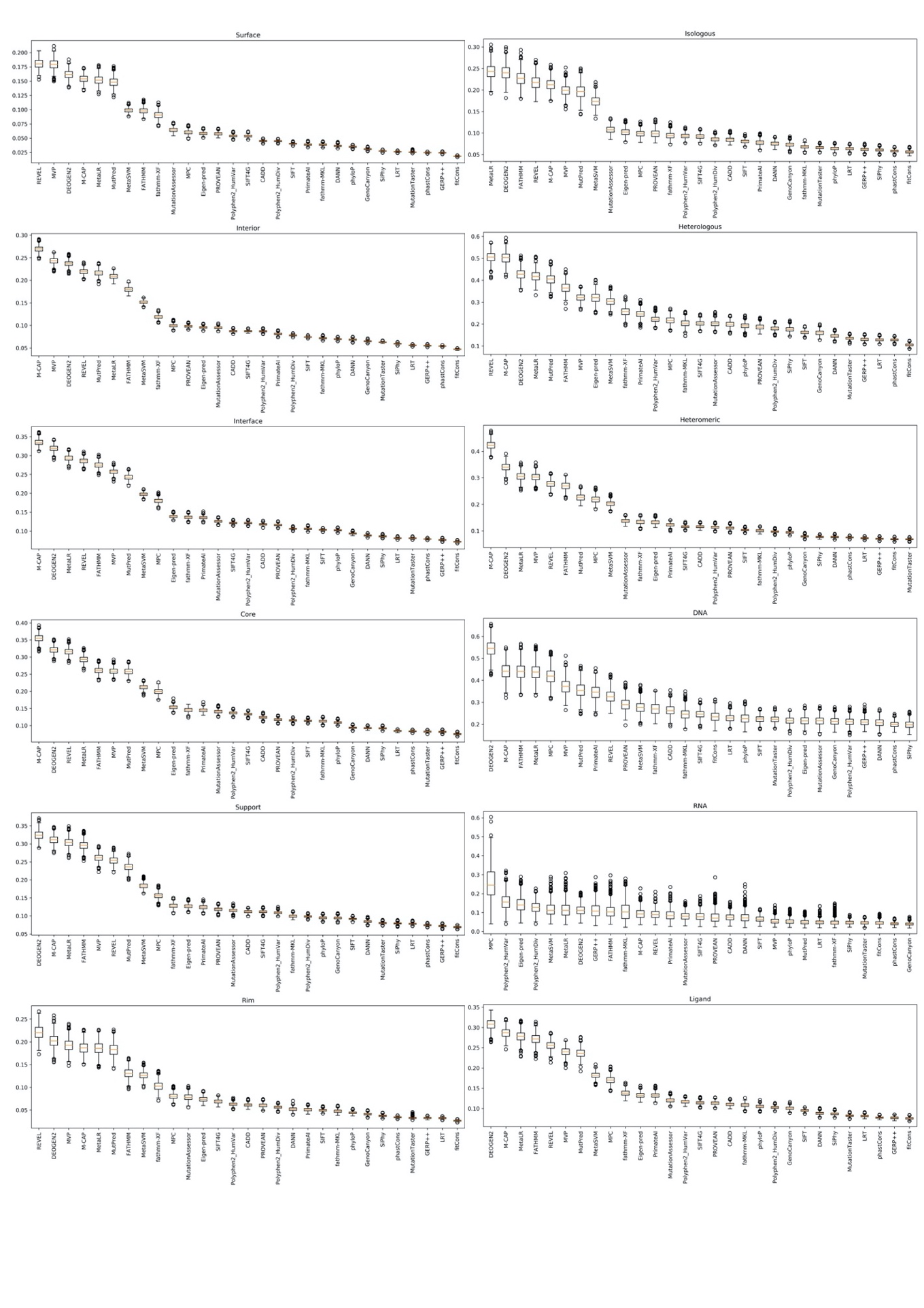


**Figure L: Distribution of bootstrapped precision recall AUCs for variant effect predictions at different protein locations and interface types.** Pathogenic and putatively benign datasets were independently re-sampled 1000 times with replacement. Only mutations with all 29 predictions were included in this analysis.
