# Supplementary material for: The properties of human disease mutations at protein interfaces
Source: PLoS Comput Biol. 2022 Feb 4;18(2):e1009858. doi: 10.1371/journal.pcbi.1009858 (PMC8849535; doi:10.1371/journal.pcbi.1009858)
Supplement: Attachment — Submitted filename: PLOS_One _review.docx [file pcbi.1009858.s005.docx]

The paper by Livesey et al. addresses the problem of variants at protein interface and how this varies according to different types of interfaces. The manuscript is interesting and well written. However, there are few aspects in the methodology that need clarification.

Major comments:

- The authors have included all variants reported in GnomAd in the “putatively benign” dataset. Although it is true that no data from individuals with rare diseases are included in GnomAD, it has to be noted that the great majority of GnomAd variants are extremely rare in the population and they could be damaging variants whose role in human pathology has not been reported yet. The benign dataset should only include GnomAd variants that are common in the population e.g. with MAF >=1. In alternative, the authors could include a sub analysis by considering separately the two datasets: putatively benign with MAF>=1 and putatively benign MAF <1. One would in fact expect most of the very rare variants in GnomAd to behave like the ClinVar damaging variants.
- Why did the authors not select as benign the ‘benign’ and ‘likely benign’ variants from ClinVar and then enrich this set with common variants from GnomAd? Although the benign variants in ClinVar are probably already included in GnomAD.
- It is not clear how were the PDB structures selected. It appears that the authors identified human proteins used for all analyses by using a sequence identity >90%. By using this criterium the dataset of experimental structures may well include 3D structures from organisms other than human. Why was PDB not searched using the UniProt ID of human proteins? This approach would result in a real dataset of 3D structures for human proteins.
- The authors should present the number of complexes in each set of interface type. Did they have, in each set, a number of complexes large enough to draw conclusions? One would expect some sets, such as the heterologous group, to be quite small.
- The authors included in the analysis the enrichment of variants at ligand binding site. This is a very interesting point, however the way ligands were selected raises questions. The authors seem to be selecting ligand binding sites simply by analysing PDB structures. It is well known that many ligands in PDB files are not biologically relevant but are present simply because they aid crystallization. If these non-biologically relevant ligands were included in the analysis, they could bias the results of variant enrichment at ligand binding sites. The authors should clarify this point.

Minor:

- I am not familiar with AREIMOL, but can the program be used to identify residues making contacts with small ligands, such as metal ions?
- Do the authors have any data on the enrichment of pathogenic variants in transient versus stable complexes? Discerning transient versus stable complexes is not easy and I am not sure there are databases reporting this information.
- When performing the analysis on distance from interface, the authors divide the variants in 30 bins. Is the variant dataset redundant? Can the same variant appear in different bins or twice in the same bin (for example in cases where a residue harbouring a variant is in proximity to two different interfaces)?
- For clarity, the definition of “putatively benign variants” which is in the Results section, should also be included in Methods, similarly to what done for pathogenic variants.
- Did the authors include NMR structures in the dataset of experimental 3D structures? And is so, which structure from the ensemble was selected for the analysis?
- The definition of SASA should be also reported in Methods not just in Results.
- With regards to the statistics and the calculation of the OR, I am not familiar with scipy.stats.fisher_exact()function from the python scipy package, but it seems odd to use a fisher test package when dealing with extremely large datasets.
- Is the residue dataset redundant or non-redundant? In other words, in the case of isologous complexes the same protein X would be represented by chains A and B in the PDB file. If the calculation of interface residues is performed at chain level, the same set of interface residues would be counted twice: once on chain A and once of chain B, thus resulting in a redundant dataset. Can the authors clarify this?
- In Methods, can the authors expand on the AAindex database? how are the ‘scale’, ‘direction’ and ‘effect size’ of amino acid changes calculated? Are these values retrieved directly from AAindex?
- In the figures, the p value for enrichment could be presented.
- The resolution of most figures appears low, but this is probably just because of the way different files were collated during the submission. Please double check the original files.
