# Supplementary material for: The properties of human disease mutations at protein interfaces
Source: PLoS Comput Biol. 2022 Feb 4;18(2):e1009858. doi: 10.1371/journal.pcbi.1009858 (PMC8849535; doi:10.1371/journal.pcbi.1009858)
Supplement: Attachment — Submitted filename: Reviewer response.docx [file pcbi.1009858.s006.docx]

**Summary of changes**

1. We added several new supplemental figures and tables in response to the specific comments of the reviewers. Previous supplemental figures and tables have been renumbered when necessary.
   a. Fig S1 - showing enrichment of pathogenic variants at cognate and non-cognate ligands
   b. Fig S2 - repeating some of our analyses using different sets of ‘benign’ variants
   c. Fig S4 – testing for disease mutation enrichments in groups of proteins with different biological functions
   d. Fig S5 – looking at the effects of interface size on disease enrichment
   e. Fig S6 – controlling for protein length and number of variants in our dataset
   f. Fig S9 – patterns of amino acid substitutions from cysteine and to proline
   g. Fig S12 – comparison of variant effect predictors using average precision score
   h. Table S1 – contains in-depth information about every enrichment calculation
   i. Table S3 – contains summary statistics for the distributions plotted in Fig 6
2. We added a new subsection to the Results called “Interface enrichment vs complex symmetry, biological function and interface size”, containing some of our new analyses, plus our previous results on complex symmetry.
3. We added a new paragraph to the Conclusions addressing possible issues with data circularity.
4. We expanded on and clarified our Methods in a few places
5. We made minor grammatical changes throughout the manuscript for improved clarity.

**Response to reviewers’ comments**

**Reviewer 1**

*The authors have included all variants reported in GnomAd in the “putatively benign” dataset. Although it is true that no data from individuals with rare diseases are included in GnomAD, it has to be noted that the great majority of GnomAd variants are extremely rare in the population and they could be damaging variants whose role in human pathology has not been reported yet. The benign dataset should only include GnomAd variants that are common in the population e.g. with MAF >=1. In alternative, the authors could include a sub analysis by considering separately the two datasets: putatively benign with MAF>=1 and putatively benign MAF <1. One would in fact expect most of the very rare variants in GnomAd to behave like the ClinVar damaging variants.*

*Why did the authors not select as benign the ‘benign’ and ‘likely benign’ variants from ClinVar and then enrich this set with common variants from GnomAd? Although the benign variants in ClinVar are probably already included in GnomAD.*

Using only the gnomAD common variants (> 1% MAF) reduces the size of our putatively benign dataset to only 2184 variants from almost 500,000. This dramatically reduces the power of many of our analyses, particularly those where small subsets of the data are used. However, to address this, we have now performed the analyses presented in Figure 1 and Figure 2 using the common and rare gnomAD variants separately, and also the ‘benign’ and ‘likely benign’ variants from ClinVar. This included in the new Fig S2. While somewhat greater levels of enrichment were observed when using the common or ‘benign’ alleles alone, very similar trends persist even when using rare alleles exclusively. We believe this justifies our choice to make use of the full gnomAD dataset for subsequent analyses where sub-setting reduces the available power. We have added a new paragraph discussing this in the section “Enrichment of disease mutations at different types of protein interfaces”.

*It is not clear how were the PDB structures selected. It appears that the authors identified human proteins used for all analyses by using a sequence identity >90%. By using this criterium the dataset of experimental structures may well include 3D structures from organisms other than human. Why was PDB not searched using the UniProt ID of human proteins? This approach would result in a real dataset of 3D structures for human proteins.*

By including structures with close homology to human sequences, we can substantially increase the size of our structural dataset. This is quite standard practice in structural bioinformatics analyses. We consider only regions with >90% sequence identity to human, which means we are only considering homologues that are very close to human. Moreover, we are only include human mutations mapped to structure at residues that have the same amino acid as the human sequence, as well as the same amino acid as the human sequence at both adjacent residues in the structure. We have now clarified this in the Methods.

*The authors should present the number of complexes in each set of interface type. Did they have, in each set, a number of complexes large enough to draw conclusions? One would expect some sets, such as the heterologous group, to be quite small.*

We have now added a new Table S1 with in-depth information about every enrichment calculation including the number of genes within each category. Figure legends have been updated to refer to this table where relevant. We think we have enough mutations from enough complexes to have high confidence in the enrichments we see. For example, the heterologous group has 1128 pathogenic mutations. Only the RNA group is small, with 68 pathogenic mutations, and we do discuss the limited confidence in these specific results in the manuscript.

*The authors included in the analysis the enrichment of variants at ligand binding site. This is a very interesting point, however the way ligands were selected raises questions. The authors seem to be selecting ligand binding sites simply by analysing PDB structures. It is well known that many ligands in PDB files are not biologically relevant but are present simply because they aid crystallization. If these non-biologically relevant ligands were included in the analysis, they could bias the results of variant enrichment at ligand binding sites. The authors should clarify this point.*

In order to investigate the impact that non-relevant ligands may be having on our analysis, we have now used FireDB ligand classifications to split all ligands into ‘cognate’ and ‘non cognate’ and analysed each separately. Cognate ligands do indeed show the greatest disease enrichment, although the enrichment at non-cognate ligand interfaces was still significant. This may be due to drug molecules binding at conserved sites. We have added a new Fig S1 showing the disease enrichment when ligands are split into cognate and non-cognate, and discuss these results in the section “Enrichment of disease mutations at different types of protein interfaces”.

*I am not familiar with AREIMOL, but can the program be used to identify residues making contacts with small ligands, such as metal ions?*

Yes, AREAIMOL absolutely does identify contacts with all small molecules, including metal ions.

*Do the authors have any data on the enrichment of pathogenic variants in transient versus stable complexes? Discerning transient versus stable complexes is not easy and I am not sure there are databases reporting this information.*

We have no direct information regarding the transience of interactions. However, previous work (<https://pubmed.ncbi.nlm.nih.gov/16104020/>) has shown that transient interfaces tend to be smaller than more permanent interfaces. We have therefore now calculated the enrichment of disease mutations within interface residues in bins of different interface size. We observe some interesting results, with disease mutations clearly enriched in large homomeric interfaces, but not for heteromeric interfaces. In contrast, disease mutations are enriched in small DNA interfaces. These results are included in a new Fig S5, and discussed in the new section ‘Interface enrichment vs complex symmetry, biological function and interface size’.

*When performing the analysis on distance from interface, the authors divide the variants in 30 bins. Is the variant dataset redundant? Can the same variant appear in different bins or twice in the same bin (for example in cases where a residue harbouring a variant is in proximity to two different interfaces)?*

The same variant can only ever appear once when calculating proximity. The interface centroid is only ever for the closest interface, where multiple interface are present in the structure. The same is true for the other distance metrics. Only the closest interface to the variant (by any metric) is considered in each case. We have now clarified this in the Methods section.

*For clarity, the definition of “putatively benign variants” which is in the Results section, should also be included in Methods, similarly to what done for pathogenic variants*

We have now added the ‘putatively benign’ definition to methods under “ClinVar and gnomAD databases”.

*Did the authors include NMR structures in the dataset of experimental 3D structures? And is so, which structure from the ensemble was selected for the analysis?*

We do include some NMR structures, although overall they represent quite a small fraction of our dataset. Where we map a variant to an NMR structure, we simply take the first structure from the ensemble. We have now updated the Methods to clarify this.

*the definition of SASA should be also reported in Methods not just in Results.*

We have now added the definition of SASA to the “Solvent Accessible Surface Area” section of the Methods.

*With regards to the statistics and the calculation of the OR, I am not familiar with scipy.stats.fisher_exact()function from the python scipy package, but it seems odd to use a fisher test package when dealing with extremely large datasets.*

The resources we have found tend to state that Fisher’s is preferred compared to the Chi-squared approximation, where computationally possible, despite a tendency to be inefficient time-wise for larger datasets.

*is the residue dataset redundant OR non-redundant? in other words, in the case of isologous complexes the same protein X would be represented by chains a and B in the PDB file. If the calculation of interface residues is performed at chain level, the same set of interface residues would be counted twice: once on chain a and once of chain B, thus resulting in a redundant dataset. Can the authors clarify this?*

The dataset of variants is non-redundant both for the main analyses and for the interface proximity analysis. In the main analyses each variant is mapped to only one residue from one chain of a sinle protein structure. In the interface proximity analysis, one calculation is performed per variant, only in its mapped chain. Only interfaces on this chain can count as being ‘in proximity’ to the variant. We have now added additional clarification to ‘PDB’ and ‘3D complex residue distance calculations’ sections in the Methods.

*in Methods, Can the authors expand on the AAindex database? how are the ‘scale’, ‘direction’ and ‘effect size’ of amino acid changes calculated? are these values*

These calculations are mostly covered under the ‘Property deltas’ section of the Methods. In short, the difference between the wild-type and mutant properties were calculated for all variants. The significance of this difference or ‘delta’ was calculated between the pathogenic / putatively benign datasets using a student’s t-test and effect size by Hedges g. Poor effect sizes were observed so directionality was removed from the deltas by taking the absolute values (aka the scale without the direction). This greatly improved the effect sizes observed. We have now updated the ‘property deltas’ section in the Methods to improve clarity.

*in the figures, the p value for enrichment could be presented.*

The range of p-values we have are a bit difficult to display nicely in figures using any of the usual methods. Instead, we have produced a new Table S1 containing in-depth information about all figures using odds ratio calculations including p-values, and made reference to the table in all relevant figure legends.

*The resolution of most figures appears low, but this is probably just because of the way different files were collated during the submission. Please double check the original files.*

Our original source files are high resolution, and we will try to avoid any problems with conversion in this resubmission.

**Reviewer 2**

*There might be some kind of circular logic in the analysis, which is related to the difficulty of defining pathogenic variants. The paper uses ClinVar data to define pathogenicity, which are likely influenced/predicted by variant effect prediction tools, and many tools might have used features correlated with the protein interface to train their classifiers. Therefore, the protein interfaces might have already been considered as a factor explicitly/implicitly to define pathogenicity. We need to keep this potential circular logic in mind when interpreting the analysis results. The authors might discuss this in the discussion or conclusion section.*

That is a very interesting point, and not something that we had considered before. Overall, we judge it to be fairly unlikely that this strongly influences our results, based on the fact that relatively few predictors (particularly the popular ones) make use of interface features. It is however a possibility that should be noted. We have now added a section to the conclusions discussing the caveats of using gnomAD and ClinVar, including the danger of predictor circularity.

*The analysis pools all genes/proteins together and do the enrichment analysis using Fisher’s exact test. We need to be cautious about potential confounding factors or heterogeneity ignored when pooling all genes together. To be more convincing, it would be helpful to stratify the genes/proteins into different groups and do the analysis within each group to make sure the patterns observed can still hold. Here are some suggested stratifications: 1) stratify genes into bins of different gene length; 2) stratify genes into bins of different number of variants; 3) stratify genes by whether they are transcription factors or not.*

We used the suggested categories as well as a couple of other functional groupings (catalytic activity and transporter activity) that gave decent numbers of variants in both classes. Our results indicate similar patterns for all of the analyses, with expected exceptions (i.e. no DNA interfaces in transporter proteins). We have now added new Figs S4 and S6 that show these comparisons, and have added paragraph under “Interface enrichment vs complex symmetry, biological function and interface size”, describing these analyses.

*Figure 6 and related explanation in the manuscript is not easy to understand, not intuitive to show the differences between different location types. We don’t know how statistically significant of these differences as described in the main text. For example, a “narrow” distribution is hard to define.*

We have added some summary statistics for the distributions into the new Table S3. We have also included a more helpful explanation of what each observation means for the difference between the various protein locations in Figure 6.

*In the section: Performance of variant effect predictors on interface mutations, the ROC statistics is used. For data sets with very unbalanced positive & negative samples, the precision-recall curve might be more informative. Can the authors add the comparisons using the precision-recall AUC?*

Unfortunately, precision-recall is not suitable for the comparisons we are interested in. Our main concern is not in the relative performance of different predictors, but in the relative performance of individual predictors between different protein locations and interface types. Because we have widely varying numbers of pathogenic and benign mutations across these different groups, we cannot make a direct comparison between different locations and interface types with precision recall. ROC AUC does not have this issue as all positives/negatives are treated equally.

We can use precision recall to make a comparison between different predictors, when controlling for location or interface type, as the number of mutations will be the same for each predictor. However, we do not think that this is particularly informative as some of the predictors will have been trained on disease mutations from our dataset, and the results are almost certain to be influenced by overfitting. We have added a new Figure S12 showing the results of precision-recall analysis from all predictors, for each protein location or interface type group. We have also added a new paragraph to the ‘Performance of variant effect predictors’ section discussing this result, addressing the caveat that the comparisons between predictors are not necessarily reliable.

*Figure 5 shows high enrichment for mutations away from cysteine and mutations to proline. Strictly speaking, a mutation is defined by both the “from” and “to”. Can the author also show the enrichment results of all mutations from cysteine? Similarly, the enrichment results of all mutations to the damaging proline? Is there a specific dominating mutation pattern or there is an almost even enrichment for all patterns?*

The issue with subsetting the data into all possible amino acid substitutions is that the analysis loses power (which is why we moved on to the property analysis from this). However, we have now included the specific mutation patterns for cysteine and proline in the new Fig S9, and discuss this in the section “Properties of amino acid substitutions at interfaces”. The mutation pattern of cysteine is actually quite interesting in that is shows no difference between the potential mutants at surface positions, but bulky mutants are more pathogenic at other locations.

*When collecting variants from gnomAD, is there an allele frequency threshold? Or matching the variant allele frequency in ClinVar?*

There was no allele frequency threshold for any of our source data. Reviewer 1 posed similar concerns, so we performed the first analyses using a MAF threshold of 1% in the new Fig S2.

*Figure 5 legend: It should be odds ration instead of log odds ratio*

Indeed it should be, the error was also present in the legend of figure 4. We have now corrected the legends of Figures 4 & 5.

*The following is not correctly referenced: Vitkup et al, 2003*

This has now been fixed.

**Reviewer 3**

*I would like to suggest authors to include most deleterious mutations (Experimentally proved) and the least deleterious mutations in the current data set.*

We believe this point is suggesting that we add filtering criteria to our datasets (along with similar points from Reviewers 1 and 2). In response to them, we looked at different subsets of the gnomAD dataset by minor allele frequency, and also considered the ClinVar benign and likely benign variants. For the ClinVar disease variant dataset, we already filter out variants with conflicting interpretations. Further reducing the size of the disease variant dataset would greatly reduce the power of our analyses.

*Any insight on the mutation(s) altering interface (protein-RNA, protein-protein, Protein-DNA and protein-ligand ect) simultaneously.*

We are unsure what this comment is referring to. If this is asking whether variants can be counted twice (as in Reviewer 1’s comments), then all variants are only counted once for the figures. There may be a very small fraction of mutations that occur at the edge of two different types of interfaces (e.g. protein:protein and protein:DNA), but the numbers will be too small for us to do any meaningful analysis.

*An explanation required on location (near, far ,very far from the interface) of the mutation site and its disease association.*

Our explanation is partially in the introduction to the section, partially after the presentation of the figure and partially in the conclusions section. We have added some further clarification to the section “Enrichment of disease mutations in proximity to interfaces”.

*Does the author point out any particular mutation /type affecting various interactions or the frequent type of mutation?*

We are unsure to what exactly the reviewer is referring here. We present enrichments of the amino acids that are mutated from/to in Figure 5 and S8. We haven’t subsetted this by all possible types of amino acid substitutions, as there are too many possible substitutions relative to the size of our dataset, but we do now include this for proline and cysteine substitutions in the new Fig S9, in response to Reviewer 2.

*It will be good a information if authors incorporate frequency of mutation and the disease association. Second information on the nature of amino acid mutation and the disease association.*

The first part was covered in our response to reviewer 1 on allele frequency in the gnomAD data. We are unclear about the second part as once again, this is covered in Figure 5/S8.
