# Supplementary material for: The properties of human disease mutations at protein interfaces
Source: PLoS Comput Biol. 2022 Feb 4;18(2):e1009858. doi: 10.1371/journal.pcbi.1009858 (PMC8849535; doi:10.1371/journal.pcbi.1009858)
Supplement: Attachment — Submitted filename: Reviewer response.docx [file pcbi.1009858.s007.docx]

**Response to reviewers’ comments**

**Reviewer 2**

*The authors addressed my questions well except the following one:
It is not the precision score metric I suggested but the area under the curve (AUC) of the precision-recall curve. The ROC curve uses the sensitivity and specificity as x and y axis, the precision-recall curve uses the precision and the sensitivity as the x and y axis. We can calculate the AUC of either the precision-recall curve or the ROC curve, as long as positive and negative samples are available and a score is defined for each sample. The should both work for the purpose of comparing the performance stratified by the protein locations and interface types. Usually the AUC of the precision-recall curve gives us another view of the performance especially when the positive-negative ratio is far away from 1. The authors can check the REVEL paper where both AUC of ROC curve and precision-recall curve were calculated:* [*https://www.ncbi.nlm.nih.gov/pmc/articles/PMC5065685/*](https://www.ncbi.nlm.nih.gov/pmc/articles/PMC5065685/)

We have now replaced the precision score metric with the precision recall AUC for the analysis in Fig S12, as suggested by the reviewer. The results are nearly identical.
